# Effect of Denture Base Fabrication Technique on *Candida albicans* Adhesion In Vitro

**DOI:** 10.3390/ma14010221

**Published:** 2021-01-05

**Authors:** Avi Meirowitz, Arkadi Rahmanov, Eti Shlomo, Helena Zelikman, Eran Dolev, Nir Sterer

**Affiliations:** Department of Prosthodontics, Goldschleger School of Dental Medicine, Sackler Faculty of Medicine, Tel-Aviv University, Ramat-Aviv 69978, Israel; eintmz@tauex.tau.ac.il (A.M.); dretishlomo@gmail.com (E.S.); helenapl@gmail.com (H.Z.); eran@drdolev.com (E.D.)

**Keywords:** dentures, biofilm adhesion, *Candida*

## Abstract

Denture stomatitis is a common manifestation of oral candidiasis affecting some 65% of denture wearers. This condition is initiated by the adherence of *Candida albicans* to denture base acrylic resin. The present study aimed to test the in vitro effect of traditional and novel fabrication methods on *Candida albicans* adhesion to denture base samples. Denture based acrylic discs were fabricated using: (i) computerized milling, (ii) 3D printing, (iii) heat curing, and (iv) cold curing. Discs were tested for surface roughness (Ra), hydrophobicity (contact angle), mucin adsorption (Bradford assay), and *Candida albicans* adhesion. 3D printing significantly increased microbial cell adhesion as compared with heat curing, and computerized milling significantly decreased it. These results were associated with mucin adsorption levels rather than surface roughness. Results suggest that 3D printing may increase the risk for developing denture stomatitis, whereas computerized milling may decrease it as compared with traditional heat curing denture base fabrication.

## 1. Introduction

The prevalence of denture stomatitis among denture wearers ranges from 15% to over 70%. The etiology of denture stomatitis is multifactorial and has a number of associative factors rather than a single cause. Poor denture hygiene, pathogenic *Candida* infection, and the continual wearing of dentures appear to be the predominant associated etiological factors for denture stomatitis [1].

Candida infection results from the adhesion and proliferation of microorganisms such as *Candida albicans* onto the denture’s acrylic base and may give rise to painful inflammation of the oral mucosa adjacent to the contaminated dentures [1,2].

Acryl-based complete or partial dentures are commonly used in the treatment of edentulous patients, especially among the elderly [3]. Traditionally, the acrylic base is prepared by heat or cold curing. However, recent technological developments have introduced additional methods of fabrication, including 3D printing and computerized milling [4,5].

CAD/CAM dentures offer several potential benefits to both the clinician and the patient. The fabrication process was found to be the most accurate, less porous, and fast manufacturing time [6].

Dentures’ acrylic base most commonly comprises of poly-methylmethacrylate (PMMA), a porous material that is prone to microbial biofilm accumulation [7]. Microbial adhesion is the first step in biofilm formation. It is mediated by the adsorption of salivary proteins and mucins pellicle onto the surface [8]. This microbial biofilm may harbor opportunistic pathogens such as *Candida albicans* that have been implicated in precancerous lesions [9] and oral infections [2]. *Candida*-infected leukoplakia indeed relates to a higher rate of malignancy [10]. *Candida* infections may play a role in the development of oral epithelial dysplasia and neoplasia, and there is an association between oral epithelial dysplasia and harboring *Candida* species in the oral cavity [11]. However, it is yet to be established whether *Candida* infections are indeed able to cause oral cancer.

Various physical properties, including surface roughness, hydrophobicity, and chemical composition, have been associated with microbial adhesion to denture surfaces [12,13]. The aim of the present study was to test the effect of traditional and novel denture base fabrication methods on *Candida albicans* adhesion and surface properties.

## 2. Materials and Methods

Six denture base acrylic discs (12 mm in diameter and 2 mm thickness) were prepared using four fabricating techniques as follows: (i) milling (CAD\CAM, Vira Vionic Base, VITA, Bad Säckingen, Germany), (ii) 3D printing (FreePrint denture, Detax, Ettlingen, Germany) (iii) hot curing (Novodon, Novodent ETS, Eschen, Liechtenstein), and (iv) cold curing (NOVO press plus, Novodent ETS, Eschen, Liechtenstein).

CAD/CAM PMMA-based specimens, designed using a computer program and then manufactured by milling (Yenamak D40, Yenadent Ltd., Istanbul, Turkey). For conventional PMMA (cold and hot cure), acrylic resin discs were prepared according to the manufacturer’s instructions. The specimens were fabricated from an acrylic resin denture base material using a conventional flasking and pressure-pack technique.

Finishing and polishing were carried out by a single operator on one side of the discs. Finishing was performed by an electric motor handpiece using a silicon carbide bar (NTI Kahla, Germany) and applied for 15 s. Polishing was performed using a hard brush and pumice paste, followed by a soft brush and universal polishing paste (Ivoclar, VivaDent, Schaan, Liechtenstein). Following polishing, the discs were rinsed with water and tested for surface properties and microbial adhesion, as described below.

Samples were mounted on aluminum stubs and sputter-coated with gold. 3D images were constructed using scanning electron microscopy (SEM, JSM-IT100, Jeol, Tokyo, Japan) in 1000× magnification. Surface roughness (Ra) was measured using image analysis software (Alicona, Bruker, Besançon, France, Mex Version 6.2).

A contact angle test was performed to evaluate the hydrophobic properties of the tested discs. The contact angle was measured using a goniometer (model 100, Ramé-Hart, Succasunna, NJ, USA) by placing a drop of deionized water (10 μL) on the leveled disc, measuring the contact angle on both sides of the drop and recording the mean result.

Pig gastric mucin (Sigma, Rehovot, Israel) was dissolved in PBS (1% *w*/*v*). A drop (40 μL) of filtered mucin (0.22 mm, stericup, Millipore, MA, USA) was placed on each of the tested discs for 3 min at room temperature [14]. Discs were washed three times with saline to remove any non-adhered mucin. Discs were placed on the bottom of a 24 wells plate, and the wells were added with 600 μL of Bradford reagent (Bradford 1976) [15]. The reagent was allowed to react for 10 min then transferred to a 96 well plate and read at 600 nm.

Discs were mucin coated as stated above and placed at the bottom of a 24 wells plate. Wells were added with 1 mL of *Candida albicans* (ES 58919, Clinical isolate, kindly provided by Prof. Segal Ester) [16] suspension (0.4 OD, equivalent to 10^6^ microbial cells per mL) in BHI broth and incubated for 4 h at 37 °C. Following incubation, the discs were washed three times with saline to remove any non-adhered microbial cells. Samples were fixated in glutaraldehyde (2.5%), dehydrated in a series of ethanol solutions (30–100%), and desiccated. Following desiccation, samples were gold-sputtered and adhered microbial cells were quantified using scanning electron microscopy (SEM, JSM-IT100, Jeol, Tokyo, Japan) by analyzing the digital images using a morphometric software (ImageJ, NIH, Version 1.52a).

To compare the effect of the different fabrication techniques on surface properties and microbial adhesion ANOVA was applied with post-hoc pairwise comparisons according to Dunnet and Scheffe. Pearson correlation coefficient was used to calculate the level of association between microbial adhesion and the various surface parameters. Tests applied were two-tailed, and *p* ≤ 0.05 was considered statistically significant. Experiments were conducted in six replicates. 

## 3. Results

**Figure 1:**
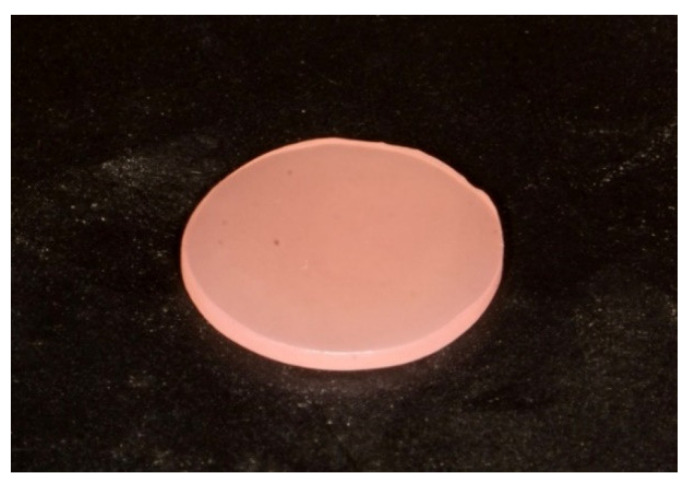
Image of milled denture base acrylic discs (CAD\CAM, Vira Vionic Base, VITA).

Results of the acrylic discs surface roughness (Ra) in the various test groups are presented in Figure 1. All the tested surfaces were smooth and yielded Ra values ranging between 0.2–0.3 μM. Results show that the surface roughness of the cold cure acrylic group was slightly higher than the other groups (*p* < 0.03).

**Figure 2:**
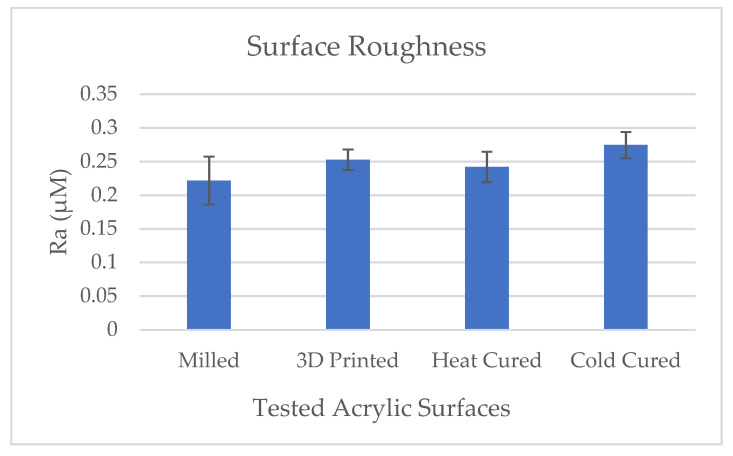
Effect of denture acrylic base fabrication method on surface roughness. Mean results (±standard deviation) are presented as Ra (µM) measured using profilometric software (AliconaTM).

Results of the acrylic discs’ surface hydrophobicity in the various test groups are presented in Figure 2. Results show that the surface hydrophobicity (contact angle) of the cold cure acrylic group was significantly higher as compared with the other tested groups (*p* < 0.001).

**Figure 3:**
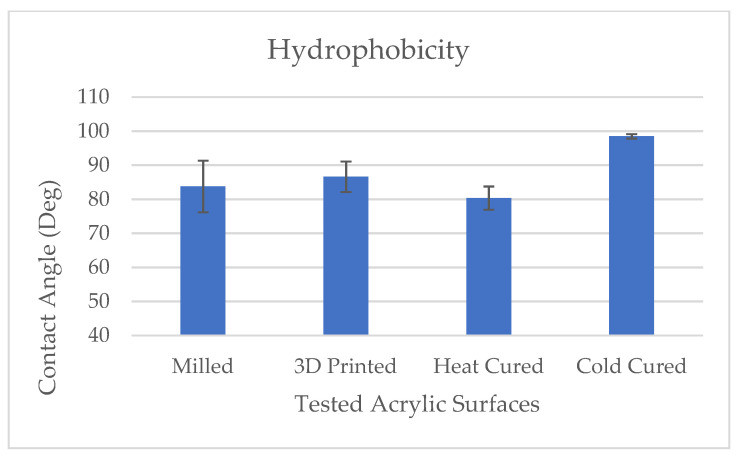
Effect of denture acrylic base fabrication method on surface hydrophobicity. Mean results (±standard deviation) are presented as the contact angle measured using a goniometer (Ramé-Hart).

Results of the mucin adsorption to the various test groups are presented in Figure 3. Results show that mucin adsorption onto the acrylic surfaces was significantly higher in the 3D printed samples as compared with the other tested groups (*p* < 0.001).

**Figure 4:**
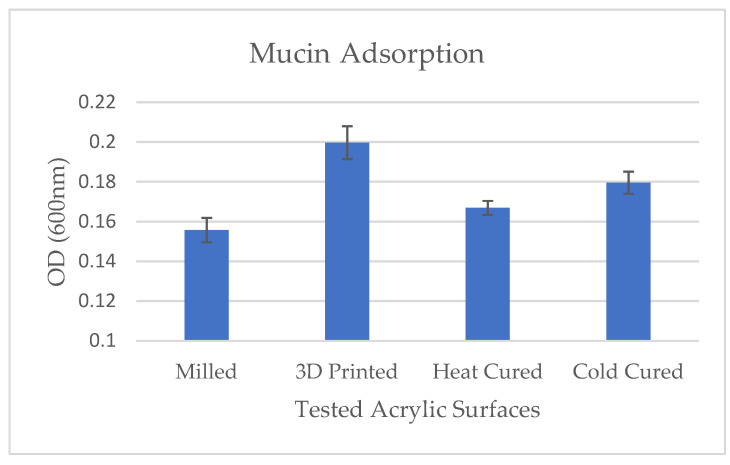
Effect of denture acrylic base fabrication method on mucin adsorption. Mean results (±Scheme 600 nm).

**Figure 5:**
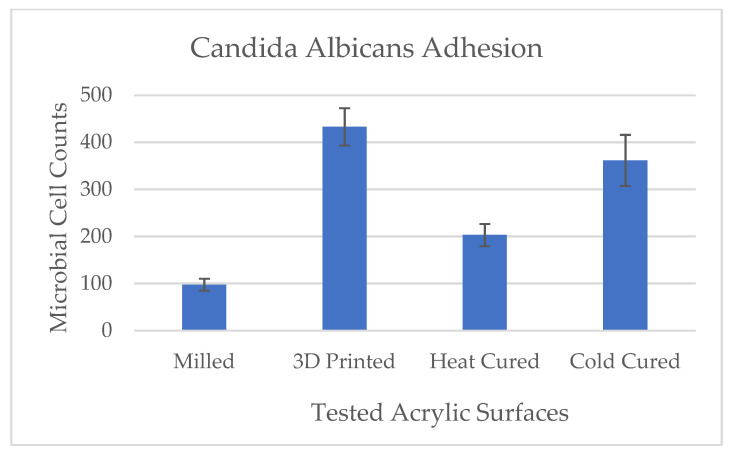
Effect of denture acrylic base fabrication method on *Candida albicans* adhesion. Mean results (±standard deviation) are presented as microbial cell counts using morphometric analysis software (ImageJ).

Results of the microbial adhesion to the various test groups are presented in Figure 4 and Figure 5. Results show that the microbial cell counts adhered to the 3D printed discs were significantly high as compared with the heat-cured samples whereas the milled samples showed significantly low counts (*p* < 0.001). Microbial adherence to cold cure acrylic discs were also significantly higher as compared with the heat cure discs (*p* < 0.001).

Levels of association between the adhered microbial cell counts and the various surface parameters are presented in Table 1. Pearson correlation analysis showed significantly high association between microbial cell counts and the levels of mucin adsorption onto the tested surfaces. Furthermore, hydrophobicity was moderately associated with all the tested parameters.

**Figure 6:**
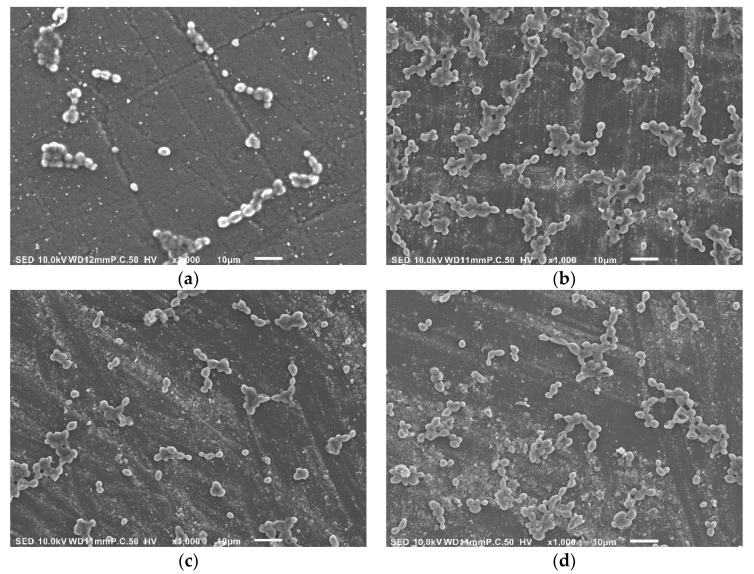
SEM images (×1000; JSM-IT100, Jeol) following microbial adhesion assay for the denture base acrylic samples fabricated using: (**a**) computerized milling, (**b**) 3D printing, (**c**) heat curing, and (**d**) cold curing.

In the present study, we tested the effect of the fabrication method on the adhesion of *Candida albicans* to denture base acrylic resin samples using two traditional production methods (heat and cold cure) and two novel methods (3D printing and computerized milling). Results showed that manufacturing denture base acrylic resin discs using 3D printing significantly increased *Candida albican’s* adhesion as compared with the traditional method of heat curing, whereas computerized milling significantly decreased the microbial adhesion (Figure 6). These results were highly associated with the level of mucin adsorbed to these surfaces. Cold cured samples, on the other hand, showed significantly high counts of microbial cell adhesion concomitantly with a significant increase in surface roughness but without a significant increase in mucin adsorption.

Previous studies showed that mucin is an important mediator in the adhesion of *Candida albicans* to surfaces [7,8]. Mucins affect the process of Candida biofilm formation and the amount and rigidity of formed biofilm [17]. They are able to adhere onto hydrophobic surfaces via their hydrophobic protein portion of the macromolecule, thus leaving their carbohydrate side chains available as a binding site for microorganisms [18]. In the present study, surface hydrophobicity was moderately associated with both mucin adsorption and microbial adhesion, whereas surface roughness did not. In contrast, other studies reported that surface roughness was a major factor in microbial adhesion to denture base acrylic resin [11,19,20]. These differences may be due to the use of mucin as a mediator for this process.

Denture stomatitis is a condition that is hard to treat. Candida biofilm formed on uncoated acrylic resin exhibits resistance to removal [17]. Therefore, its prevention is a high priority. Various researchers have attempted to incorporate antimicrobial nano-particles into the denture base acrylic resin [21,22,23]. However, these may affect the material’s physical properties, and their effectiveness over time is not yet established. Other researches have used various coating materials to prevent microbial adhesion [24,25,26]; of course, these may also wear off over time. Therefore, the quest for better materials and fabrication methods is still on the way.

Several research studies have suggested that the materials’ surface roughness is an important factor affecting the adhesion of *C. albicans* [27]. Studies reveal that the initial adhesion of microorganisms generally begins in the pores of rough surfaces, which are known to provide protection against shear forces and provide time for irreversible adhesion of microbial cells to the surface [28]. Murat et al. showed that CAD/CAM PMMA-based polymers have less surface roughness and *Candida* adhesion when compared to conventional polymerized PMMA [21].

Within the limitations of this in vitro study, its results suggest that 3D printing of denture base may enhance microbial adhesion compared with traditional heat curing fabrication, thus increasing the risk of developing denture stomatitis and other *Candida albicans* related problems whereas computerized milling may decrease those risks. Further research should investigate the effect of the percentage of denture materials polymerization on bacterial adherence.

## Figures and Tables

**Table 1:** Pearson correlation coefficients between microbial adhesion and surface parameters.

	Mucin Adsorption	Hydrophobicity	Surface Roughness
Microbial cell counts	r = 0.812 *p <* 0.001 ^1^	r = 0.429 *p =* 0.036 ^1^	r = 0.330 *p =* 0.114
Mucin adsorption		r = 0.424 *p =* 0.038 ^1^	r = 0.338 *p =* 0.105
Hydrophobicity			r = 0.530 *p =* 0.007 ^1^

^1^ Indicates data statistically significant.

## Data Availability

Data sharing not applicable.
